# Supplementary material for: The Evolution and Characterization of the RNA Interference Pathways in Lophotrochozoa
Source: Genome Biol Evol. 2024 May 7;16(5):evae098. doi: 10.1093/gbe/evae098 (PMC11114477; doi:10.1093/gbe/evae098)
Supplement: evae098_Supplementary_Data [file evae098_supplementary_data.zip › Supplementary_materials.zip!/selected_miRNAs/CrGi_selected_miRNAs/CrGi_NC_047564.1_20822.pdf]

Provisional ID	:	NC_047564.1_20822
Score total	:	73.2
Score for star read(s)	:	3.9
Score for read counts	:	66.4
Score for mfe	:	1.9
Score for randfold	:	1.6
Score for cons. seed	:	-0.6
Total read count	:	142
Mature read count	:	135
Loop read count	:	0
Star read count	:	7

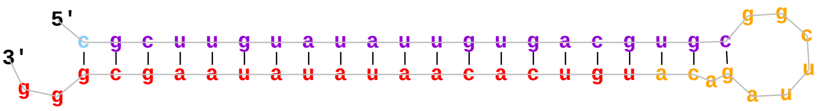

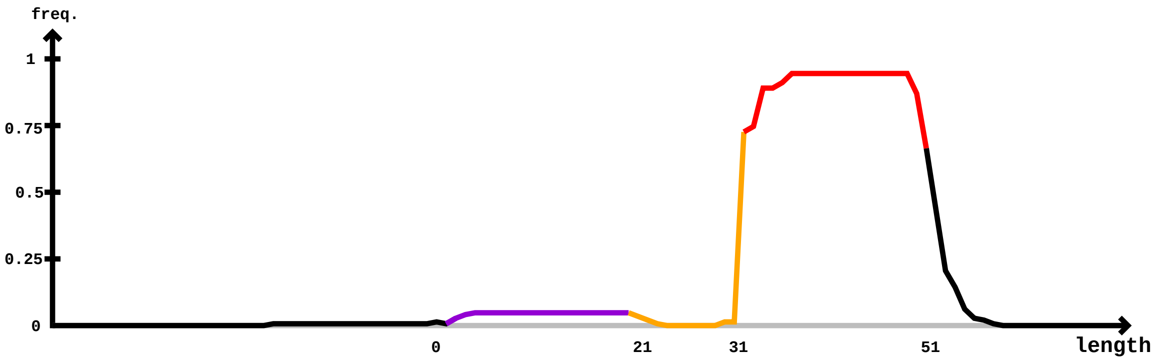


```
                                        Star                Mature
5'- cugguacauucuuaauccgaaauuccucugacucuaauccgcuuguauauugugacgugcggcuuagacaugucacaauauauaagcggggguuagaauaaaagaaaucu  -3'  obs
    cugguacauucuuaauccgaaauuccucugacucuaauccgcuuguauauugugacgugcggcuuagacaugucacaauauauaagcggggguuagaauaaaagaaaucu       exp
    ..((....(((.......)))...))((((((((....((((((((((((((((((((((......).)))))))))))))))))))))))))))))).............   reads    mm    sample
    ......................................ccgcuuguauauugugacgug...................................................   1        0     Ov2
    ......................................................................ugucacaauauaAaagcgggg...................   2        1     Ov2
    .......................................................................gucacaauauaAaagcggg....................   1        1     Ov2
    ........................................................................ucacaauauaAaagcggg....................   1        1     Ov2
    ........................................................................ucacaauauaAaagcggggg..................   2        1     Ov2
    ........................................................................ucacaauauaAaagcgggggu.................   1        1     Ov2
    ........................................................................ucacaauauaAaagcggggguu................   1        1     Ov2

    ........................................gcuuguauauugugacgug...................................................   1        0     Ov1
    .........................................cuuguauauugugacgugcgg................................................   1        0     Ov1
    ......................................................................ugucacaauauaAaagcgg.....................   4        1     Ov1
    .......................................................................gucacaauauaAaagcggggg..................   1        1     Ov1
    ........................................................................ucacaauauaAaagcggggg..................   2        1     Ov1
    ........................................................................ucacaauauaAaagcgggggu.................   1        1     Ov1
    ........................................................................ucacaauauaAaagcggggguu................   1        1     Ov1
    ..........................................................................acaauauaAaagcgggggu.................   1        1     Ov1
    ...........................................................................caauauaAaagcggggguua...............   1        1     Ov1
    ...........................................................................caauauaAaagcggggguuaga.............   1        1     Ov1

    ..........................................uuguauauugugacgugc..................................................   1        0     Mt1
    ....................................................................caugucacaauauaAaagcgg.....................   2        1     Mt1
    ......................................................................ugucacaauauaAaagcg......................   6        1     Mt1
    ......................................................................ugucacaauauauaagcgg.....................   2        0     Mt1
    ......................................................................ugucacaauauaAaagcgg.....................   9        1     Mt1
    ......................................................................ugucacaauauaAaagcggg....................   14       1     Mt1
    ......................................................................ugucacaauauauaagcUgg....................   1        1     Mt1
    ......................................................................ugucacaauauauaagcggg....................   2        0     Mt1
    ......................................................................ugucacaauauaAaagcgggg...................   12       1     Mt1
    ......................................................................ugucacaauauauaagcgggg...................   1        0     Mt1
    ......................................................................ugucacaauauaAaagcggggg..................   1        1     Mt1
    ......................................................................ugucacaauauauaagcggggU..................   1        1     Mt1
    ........................................................................ucacaauauaAaagcggggg..................   1        1     Mt1
    ........................................................................ucacaauauaAaagcgggggu.................   2        1     Mt1
```

```
                                        Star                          Mature
cugguacauucuuaauccgaaauuccucugacucuaaucc gcuuguauauugugacgugc ggcuuagaca ugucacaauauauaagcggg gguuagaauaaaagaaaucu
```

```
cugguacauucuuaauccgaaauuccucugacucuaauccgcuuguauauugugacgugcggcuuagacaugucacaauauauaagcggggguuagaauaaaagaaaucu
........................................................................ucacaauauaAaagcggggguu................   2   1   Mt1
..........................................................................acaauauaAaagcgggggu.................   1   1   Mt1
..........................................................................acaauauaAaagcggggguu................   1   1   Mt1
...........................................................................caauauaAaagcgggggu.................   1   1   Mt1
...........................................................................caauauauaagcggggguuag..............   1   0   Mt1

.....................auuccucugacucuaauc.......................................................................   1   0   Mt2
........................................gcuuguauauugugacgugc..................................................   2   0   Mt2
.........................................cuuguauauugugacgugc..................................................   1   0   Mt2
......................................................................ugucacaauauauaagcg......................   1   0   Mt2
......................................................................ugucacaauauaAaagcg......................   4   1   Mt2
......................................................................ugucacaauauaAaagcgg.....................   11  1   Mt2
......................................................................ugucacaauauauaagcgg.....................   2   0   Mt2
......................................................................ugucacaauauauaagcggg....................   3   0   Mt2
......................................................................ugucacaauauaAaagcggg....................   15  1   Mt2
......................................................................ugucacaauauauaagcggU....................   2   1   Mt2
......................................................................ugucacaauauaAaagcgggg...................   11  1   Mt2
.......................................................................gucacaauauaAaagcgggggu.................   1   1   Mt2
........................................................................ucacaauauaAaagcgggg...................   2   1   Mt2
........................................................................ucacaauauaAaagcggggg..................   1   1   Mt2
........................................................................ucacaauauaAaagcgggggu.................   4   1   Mt2
...........................................................................caauauaAaagcggggguuag..............   1   1   Mt2
```
